# The Relationship Between Basic Psychological Need Satisfaction, Fear of Missing Out, and University Student Depression: A Two-Year Follow-Up Study

**DOI:** 10.3390/bs15101379

**Published:** 2025-10-10

**Authors:** Xintong Zhao, Zixian Ren, Tao Xin

**Affiliations:** 1 School of Educational Science, Anhui Normal University, Wuhu 241000, China; zxtong@ahnu.edu.cn (X.Z.); rzx@ahnu.edu.cn (Z.R.); 2 Collaborative Innovation Center of Assessment Toward Basic Education Quality, Beijing Normal University, Beijing 100875, China

**Keywords:** basic psychological need satisfaction, fear of missing out, depression, university student, longitudinal mediation analysis

## Abstract

Previous cross-sectional studies have explored associations between basic psychological need satisfaction, fear of missing out (FoMO), and depression. However, the longitudinal nature of these relationships and their underlying mechanisms remain unclear. This study aimed to utilize longitudinal tracking methods to investigate the relationships among basic psychological need satisfaction, fear of missing out, and depression in university students. Longitudinal data collection was conducted among 750 university students (mean age = 18.12 ± 0.73) in China over two years at three time points. Participants were investigated using paper–pencil survey versions of the Basic Psychological Needs Scale, the Fear of Missing Out scale, and The Center for Epidemiological Studies Depression Scale. The results revealed that, over the two-year study period, basic psychological need satisfaction (*β* = −6.239, *p* < 0.001) among university students demonstrated a declining trend, while FoMO (*β* = 1.360, *p* < 0.001) and depression (*β* = 3.602, *p* < 0.001) demonstrated an upward trend. The initial levels and development rates of basic psychological need satisfaction directly predicted the initial levels (*β* = −0.236, *p* = 0.031) and development rates of depression (*β* = −0.144, *p* < 0.001; *β* = −0.181, *p* = 0.005). The initial level of FoMO mediated the relationship between basic psychological need satisfaction and depression (*β* = −0.132, *p* = 0.007; *β* = −0.104, *p* = 0.036), and this mediating effect did not exhibit significant gender differences. These findings help to reveal the temporal relationships among the three variables from a dynamic perspective, providing important practical guidance for mental health education in universities.

## 1. Introduction

Depression, or depressive disorder, is primarily characterized by a persistently low mood—a transient yet intense negative emotional state often triggered by stressful life events (Orsolini et al., 2020). Global Burden of Disease found that mental disorders rank among the top ten causes of health loss worldwide, with anxiety and depression being the most prevalent across all age groups and regions and depression rates showing an upward trend (Global Burden of Disease, 2021). According to the World Health Organization, it is projected to become the leading contributor to the global disease burden by 2030 (World Health Organization, 2018). University students, who are at a crucial stage of social and psychological development, often face a distinctive set of stressors (Paul & Brier, 2001). The pervasive use of social media in contemporary life increases their exposure to social comparison and identity confusion, contributing to heightened psychological pressure (Yang et al., 2024). As a group confronted with multiple developmental challenges—such as academic competition, career uncertainty, and the restructuring of interpersonal relationships—university students are particularly vulnerable to depressive symptoms (Shi et al., 2024). According to the China National Mental Health Development Report (2023–2024), depression levels peak among young adults aged 18–24 in China, significantly higher than in other age groups (depression levels reflect mean scores on depression scales, not clinically diagnosed depression). Meta-analysis results also indicate that the detection rate of depression among Chinese college students is on the rise year by year (Y. Chen et al., 2022). In mild cases, depression can impact appetite and sleep quality, while in severe cases, it may induce extreme behaviors such as self-injury and suicide (Gronemann et al., 2021). In the clinical setting, depression is more likely to develop if depressed mood is not regarded seriously and allowed to accumulate over time (Compare et al., 2014). Depression is a highly heterogeneous syndrome, exhibiting significant variation across multiple clinical manifestations (such as insomnia, suicidal ideation, and other individual depressive symptoms) and numerous biological characteristics (such as functional impairment) (Fried & Nesse, 2015; Olbert et al., 2014). Due to this heterogeneity, numerous distinct assessment tools have emerged in clinical and research settings. However, studies indicate that despite the use of various rating scales to assess depression severity, these instruments show only 40% overlap (Fried, 2017). Therefore, given the heterogeneous presentation of depressive symptoms and the current state of depression among Chinese university students, it is imperative to investigate the factors influencing depression in this population.

According to self-determination theory, basic psychological need satisfaction serves as a crucial environmental factor promoting individual mental health and positive development (Deci & Ryan, 2000). This theory posits that humans possess three innate, universal psychological needs: autonomy, competence, and relatedness (Deci & Ryan, 2012). When these needs are adequately met, individuals are more likely to exhibit positive behavioral patterns and experience vibrant well-being, thereby forming an internal foundation for resilience against psychological distress (Jovanović et al., 2024; Wei et al., 2021). Conversely, prolonged frustration of these needs tends to trigger negative psychological states and behaviors (Andrews et al., 2011). The theory further explains that high levels of need fulfillment enhance self-integration and emotional regulation abilities (Costa et al., 2015; Hope et al., 2019), directly reducing susceptibility to internalizing problems like depression. Conversely, chronic need frustration may lead to diminished intrinsic motivation and emotional dysregulation, becoming a core risk factor for depression (Deng et al., 2019). Empirical studies also support this perspective. For instance, Z. Liu et al. (2024) found that basic psychological need satisfaction directly alleviates depressive states. Similarly, Pi et al. (2024) conducted a longitudinal study of 553 Chinese university students, revealing a significant negative correlation between basic psychological need satisfaction and depression. In summary, conceptualizing basic psychological need satisfaction as a holistic protective factor holds significant implications for understanding and intervening in depression among university students. Considering the above, Hypothesis 1 is proposed in this study: basic psychological need satisfaction can significantly and negatively predict depression.

Fear of missing out (FoMO) is an anticipation–avoidance cognitive bias, a diffuse form of anxiety caused by a need to continuously be aware of what others are doing (Przybylski et al., 2013). This mindset drives people to monitor social platforms and engage in social activities in order to stay up-to-date with others (Przybylski et al., 2013). The growing usage of mobile social platforms has increased FoMO (Casale et al., 2018). Almost three-quarters of young people reported feeling uncomfortable when they thought they might miss out on something their peers were doing (Baker et al., 2016). In their daily lives, individuals who experience FoMO will frequently engage in social activities and frequently check social media (Elhai et al., 2016). This behavior will lead to a reduction in overall efficiency, a disruption in their workflow, and distraction (Alt, 2018).

Firstly, self-determination theory posits that the basic psychological needs must be satisfied in order for individuals to achieve effective self-regulation and maintain psychological well-being (Deci & Ryan, 2000). Individuals are susceptible to maladaptive cognitions and behaviors that disrupt their ordinary self-regulatory functioning if these basic psychological needs are neglected (Deci & Ryan, 2012). FoMO, a form of anxiety, is frequently induced when individuals’ fundamental psychological requirements are not met (Wan et al., 2024). There is a substantial negative correlation between the two, as research has demonstrated that a lack of self-regulation of psychological requirements is a significant factor in activating FoMO (Alt & Boniel-Nissim, 2018; James et al., 2017). Frustration of basic psychological needs can lead to elevated levels of anxiety (Vansteenkiste & Ryan, 2013), while FoMO, as a sub-type of anxiety, represents a self-regulatory dilemma arising from situational distress or the chronic frustration of psychological needs. When individuals’ basic psychological needs are frustrated, resulting in feelings of powerlessness, loss of control, and loneliness, their level of FoMO tends to increase, which can negatively affect their psychological well-being and self-regulation (Wegmann et al., 2017).

Social comparison theory suggests that individuals with FoMO are inclined to engage in upward comparisons with others, which results in a sense of relative deprivation and a sense of inferiority to others. This, in turn, triggers depression (Park & Park, 2024). In the context of Chinese collectivist culture, individuals devote a greater amount of attention to the evaluations of others and frequently establish their self-worth by comparing themselves to others (Cheng & Berman, 2012). Consequently, FoMO is more likely to provoke social comparisons that exacerbate depression (Zhao & Bai, 2025). According to research, the more FoMO, the more severe the individuals’ depression (Baker et al., 2016). This issue is exacerbated by the rapid growth of mobile social platforms, where FoMO can lead to improper interpersonal relationships and an increased risk of depression (Stead & Bibby, 2017). According to self-determination theory, when individuals’ basic psychological needs are frustrated, the individual’s self-regulation is impeded, and they are vulnerable to negative emotions (Ryan & Deci, 2000). Dhir et al. (2018) and M. Liu et al. (2024) have conducted empirical studies that demonstrate a positive correlation between the severity of depression and FoMO. Based on these analyses, Hypothesis 2 is proposed in this study: the relationship between depression and basic psychological need satisfaction may be mediated by FoMO.

Additionally, the associations between basic psychological need satisfaction and depression may vary by gender. According to gender role theory, when exposed to negative stimuli and information, men and women differ in their sensitivity to negative stimuli and their responses to them, with women being more susceptible to the effects of negative stimuli (Proverbio et al., 2009). Furthermore, compared to men, women are less skilled at using cognitive reappraisal and other emotional regulation strategies (Hamama & Hamama-Raz, 2021), while men typically demonstrate greater effectiveness and composure in emotional regulation (Nolen-Hoeksema, 2012). This suggests that women may be more inclined to use depression to cope with negative emotions and FoMO triggered by the frustration of basic psychological needs. Therefore, further research is needed to examine potential gender differences in the mechanisms underlying the relationship between basic psychological need satisfaction and depression.

Having reviewed the existing literature, it seems that the majority of studies on the above three aspects are cross-sectional (Butt & Arshad, 2021; Dou et al., 2023; Wan et al., 2024). The use of a cross-sectional research design makes it challenging to capture variable relationships that are characterized by ephemerality, and it can fail to disclose causal relationships on time series, as noted by Wen (2017). Thus, the utilization of cross-sectional data to verify mediation is logically flawed (Gan, 2014). Longitudinal studies have theoretical advantages over cross-sectional studies in causal inference by revealing individual trends and differences in change (Caruana et al., 2015). In research on the longitudinal mediating role of FoMO, the value of one or a few nodes is typically employed to represent the state of development. Consequently, the studies remain at the developmental level. To proceed, it is imperative to specify the initial level and rate of development of the mediating variable in order to ascertain which part of it is relevant (Reed-Fitzke & Lucier-Greer, 2021). Therefore, the present study focuses on the developmental trajectory of FoMO by analyzing both its baseline level and its rate of change as longitudinal mediators between basic psychological need satisfaction and depression. To this end, the study targets first-year university students and explores the developmental patterns of basic psychological need satisfaction, FoMO, and depression through three waves of follow-up data. A latent growth model is employed to investigate these trajectories, with a particular focus on the longitudinal mediating role of FoMO.

## 2. Methods

### 2.1. Participants and Procedure

The study used whole-group sampling to select freshmen from four universities in Anhui Province as the study subjects. Three group administrations were conducted on a class basis. The questionnaire was administered uniformly by the researchers. To control familiarity bias in participants due to repeated exposure, the order of scale presentation was varied across assessment waves in the study. The first administration (T1) was in November 2022, and 802 questionnaires were distributed. The second administration (T2, November 2023) returned 786 questionnaires, and the third administration (T3, November 2024) returned 750 questionnaires. A total of 52 subjects were lost for reasons such as suspension from school (attrition rate of 6.48%). A total of 750 valid subjects were eventually obtained, of whom 334 (44.5%) were boys and 416 (55.5%) were girls, with 363 (48.4%) urban and 387 (51.6%) rural students. For questionnaire collection, all participants provided written informed consent and volunteered to participate in this anonymous study; they were also informed that they could withdraw at any time during the study. The study was ethically reviewed and approved by the researchers’ organization. The screening criteria for valid subjects were that the information in the completed questionnaire was complete, there was no missing information, the responses were not regular and repetitive, the response process and time were reasonable, and there was no logical conflict between the previous and subsequent responses. The mean age at the time of the first administration of the test was 18.12 ± 0.73. The *t*-test analysis showed that there was no significant difference between the 52 subjects who were lost and the 750 valid subjects in basic psychological need satisfaction [*t*(750) = 0.82, *p* > 0.05], FoMO [*t*(750) = 0.82, *p* > 0.05] and depression [*t*(750) = 0.82, *p* > 0.05], indicating that there was no structural loss of subjects.

### 2.2. Measures

#### 2.2.1. Basic Psychological Needs Scale (BPNS)

The Chinese version of the BPNS developed by Gagné (2003) and revised by J. Liu et al. (2013) was used in this study. The BPNS consists of 19 items, which are categorized into three dimensions: autonomy, relatedness, and competence. Each item is scored on a 7-point scale, ranging from 1 for “strongly disagree” to 7 for “strongly agree”, with higher scores indicating higher levels of basic psychological need satisfaction. This scale has been widely applied to Chinese university students with good reliability and validity (Huang et al., 2024; Ling et al., 2025). Cronbach’s alpha coefficients for the scale across the three administrations in this study were 0.880, 0.903, and 0.910, respectively.

#### 2.2.2. Fear of Missing Out Scale (FoMOs)

The FoMOs compiled by Przybylski et al. (2013) and revised by Li et al. (2019) was used in this study. The revised scale demonstrated good reliability and validity when administered to university students (Li et al., 2019). This 8-item scale uses a 5-point Likert scale (1 = strongly disagree, 5 = strongly agree). Higher scores indicate greater FoMO. Cronbach’s alpha coefficients for the scale across the three administrations in this study were 0.922, 0.928, and 0.918, respectively.

#### 2.2.3. The Center for Epidemiological Studies Depression Scale (CES-D)

The depression scale developed by Radloff (1977) was used, with twenty items and four dimensions. A 4-point scale was used, with 0 representing “never or rarely” and 3 representing “almost always”. The higher the score, the higher the level of depression. The scale has been widely applied to Chinese university students and has shown good reliability (Qiu & Ye, 2024; L. Zhou et al., 2024). Cronbach’s alpha coefficients for the scale across the three administrations in this study were 0.908, 0.954, and 0.957, respectively.

### 2.3. Statistical Analyses

Data were recorded and organized using SPSS 26.0. Descriptive statistics and correlation analyses were conducted on valid data, followed by Harman’s one-factor test. Subsequent analyses were performed using Mplus 8.3. First, longitudinal measurement invariance was tested through configural invariance, metric invariance, and scalar invariance analyses. Second, latent growth models were constructed to examine the developmental trajectories of basic psychological need satisfaction, FoMO, and depression. Within these models, the intercept represented baseline levels, while the slope indicated developmental rates. Subsequently, a conditional growth model was developed to investigate the relationship between the intercept and slope of basic psychological need satisfaction and the intercept and slope of depression. Finally, a results equation model was constructed to test FoMO’s longitudinal mediating role in basic psychological need satisfaction and depression using the Bootstrap method. Gender differences in this mediating effect were examined through applying equation models to multiple group comparison results. The multivariate group comparison method proposed by Mulder and Hamaker (2021) was adopted in this study. We constructed an unconstrained model (M1, for which path coefficients were freely estimated across gender groups) and a constrained model (M2, for which path coefficients were constrained to be equal across gender groups). If the fit indices of M2 were significantly inferior to those of M1, the results indicated that there were differences between the groups. Otherwise, there were no differences.

## 3. Results

### 3.1. Common Method Bias Test

Since the data were collected from subjects’ self-reports, Harman’s single-factor test was used for common method bias. The results showed that there was a total of 13 factors with eigenvalues greater than one in T1, and the explanation rate of the first factor was 13.476%. There was a total of 12 factors with eigenvalues greater than one in T2, and the explanation rate of the first factor was 16.354%. There was a total of 11 factors with eigenvalues greater than one in T3, and the explanation rate of the first factor was 19.303%. All of them were less than the critical criterion of 40%, indicating that there was no serious common method bias in this study (H. Zhou & Long, 2004).

### 3.2. Descriptive Statistics and Correlation Analysis

The descriptive statistics for each variable are shown in Table 1. The three measurements of basic psychological need satisfaction and FoMO were significantly negatively correlated. The three measurements of basic psychological need satisfaction and depression were significantly negatively correlated. The three measurements of FoMO and depression were significantly positively correlated. In addition, basic psychological need satisfaction, FoMO, and depression were significantly positively correlated with the respective variables at adjacent time points. The results show that these variables are stable over time. Moreover, since gender was not significantly and negatively correlated with variables, in subsequent analyses conducted using Mplus 8.3, there was no need to include gender as a control variable in the model.

### 3.3. Longitudinal Measurement Invariance Test

Given the longitudinal nature of the data, longitudinal measurement invariance analyses were conducted. This study tested longitudinal measurement invariance across three time points: configural invariance, metric invariance (factor loading invariance), and scalar invariance (item intercept invariance). According to F. Chen (2007), a particular test of equivalence is considered to be passed if the differences between the individual model fit indices are less than a set threshold (ΔCFI < 0.01 and ΔRMSEA < 0.015). As shown in Table 2, all variables meet the scalar invariance.

### 3.4. Unconditional Latent Growth Model

An unconditional latent growth model was constructed for basic psychological need satisfaction, FoMO, and depression. In the table below, “Intercept” refers to mean initial status, and “Slope” refers to the mean rate of change (Mulder & Hamaker, 2021). The results showed that the models of basic psychological need satisfaction, FoMO, and depression were well fitted (Table 3). The slopes of FoMO and depression are positive, indicating a linear increasing trend of FoMO and depression among college students, and the slopes of basic psychological need satisfaction are negative, indicating a linear decreasing trend of basic psychological need satisfaction among college students. The intercepts and slopes of the three are not significantly correlated.

### 3.5. Conditional Latent Growth Model for Basic Psychological Need Satisfaction, FoMO, and Depression

**Figure 1:**
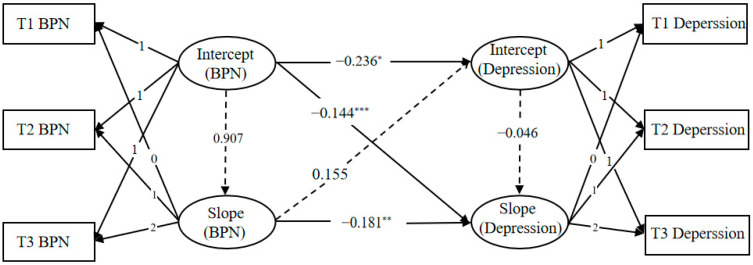
Latent growth model for basic psychological need satisfaction and depression. * *p* < 0.05, ** *p* < 0.01, *** *p* < 0.001.

A conditional latent growth model was constructed to examine the relationship between basic psychological need satisfaction and depression. As shown in Figure 1, the model demonstrated an acceptable fit to the data: χ^2^/df = 0.447, RMSEA = 0.000, CFI = 1.000, TLI = 1.009, and SRMR = 0.019. The intercept of basic psychological need satisfaction significantly negatively predicted the intercept of depression (*β* = −0.236, *p* = 0.031) and was able to significantly negatively predict the slope of depression (*β* = −0.144, *p* < 0.001). The slope of basic psychological need satisfaction significantly negatively predicted the slope of depression (*β* = −0.181, *p* = 0.005). This indicates that the higher the initial level of basic psychological need satisfaction, the lower the initial level of depression and the slower the rate of increase. The faster the rate of increase in basic psychological need satisfaction, the slower the rate of deterioration in depression.

### 3.6. The Longitudinal Mediation of FoMO

**Figure 2:**
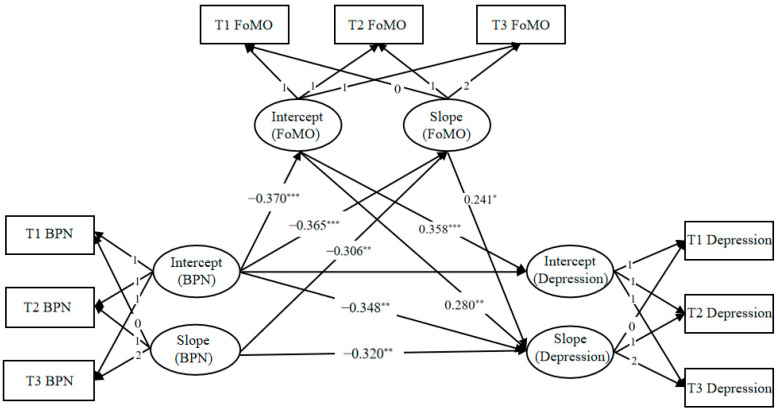
Longitudinal mediation model of FoMO. * *p* < 0.05, ** *p* < 0.01, *** *p* < 0.001.

Structural equation modeling to test longitudinal mediation was developed to examine the longitudinal mediating effect of FoMO between basic psychological need satisfaction and depression. As shown in Figure 2, the model provided an acceptable fit to the data: χ^2^(*df*) = 0.790, RMSEA = 0.000, CFI = 1.000, TLI = 1.007, and SRMR = 0.038. The direct paths for basic psychological need satisfaction, FoMO, and depression are shown in Table 4. The intercept and slope of basic psychological need satisfaction significantly negatively predicted the intercept (*β* = −0.307, *p* < 0.001) and slope (*β* = −0.365, *p* < 0.001) of FoMO, respectively. The intercept of basic psychological need satisfaction significantly negatively predicted the intercept of depression (*β* = −0.306, *p* = 0.002) and the slope of depression (*β* = −0.348, *p* = 0.002). The slope of basic psychological need satisfaction significantly negatively predicted the slope of depression (*β* = −0.320, *p* = 0.007). The intercept of FoMO significantly positively predicted the intercept of depression (*β* = 0.358, *p* < 0.001) and significantly positively predicted the slope of depression (*β* = 0.280, *p* < 0.001), and the slope of FoMO significantly positively predicted the slope of depression (*β* = 0.241, *p* = 0.039).

Based on the acceptable model fit and significant direct path of the longitudinal mediation model, the mediating role of FoMO intercept and slope was further validated using the Bootstrap method with 5000 repetitive samples, and the results are shown in Table 5. The results indicated that the intercept of FoMO mediated the relationship between the intercept of basic psychological need satisfaction and both the intercept and slope of depression. Specifically, the FoMO intercept significantly mediated the relationship between the BPNS intercept and Depression intercept (*β* = −0.132, 95% CI = [−0.231, −0.070], *p* = 0.007). The FOMO intercept significantly mediated the relationship between the BPNS intercept and the Depression slope (*β* = −0.104, 95% CI = [−0.206, −0.042], *p* = 0.036).

### 3.7. Gender Differences in Longitudinal Mediation Models

A multi-group comparative structural equation model was employed to analyze the differences in the relationships among basic psychological need satisfaction, FoMO, and depression across different genders. First, an unconstrained model (M1) was constructed, allowing the path coefficients for the male and female groups to be estimated freely. Second, a constrained model (M2) was constructed, requiring all path coefficients to be equal across genders. Through nested model comparison, if the M2 was not significantly inferior to the M1, this supported no group differences in the variable relationships. The results of the model fits are as follows: In M1, χ^2^ = 43.417, *df* = 44, CFI = 1.000, TLI = 1.001, and RMSEA = 0.000. In M2, χ^2^ = 52.669, *df* = 53, CFI = 1.000, TLI = 1.000, and RMSEA = 0.000. The nested model comparison found that M2 was not significantly inferior to M1 (Δχ^2^ = χ^2^(M2) − χ^2^(M1) = 52.669 − 43.417 = 9.252, Δ*df* = *df* (M2) − *df* (M1) = 53 − 44 = 9, *p* > 0.05), with negligible changes in the core fit index (ΔCFI = 0, ΔRMSEA = 0, ΔTLI < 0.001). Therefore, the results of the multiple group comparisons indicated that there were no significant differences in the relationship among basic psychological need satisfaction, FoMO, and depression across different genders.

## 4. Discussion

### 4.1. Trends in University Students’ Basic Psychological Need Satisfaction, FoMO, and Depression

Research has found that the satisfaction of basic psychological needs among university students is declining (*β* = −6.239, *p* < 0.001). This finding aligns with existing theoretical perspectives indicating that the satisfaction of basic psychological needs is not merely a static psychological resource but rather an ongoing dynamic adaptive process (Ryan & Deci, 2008). As their academic journey progresses, university students face numerous challenges. On the one hand, as aging and environmental changes occur, the inner needs of university students who have entered early adulthood become more diverse and complex, making the satisfaction of psychological needs more challenging (Vansteenkiste et al., 2020). On the other hand, the increasing competition in academics and practical activities has led to decreased feelings of control over new environments. As the study progressed, the participants’ academic years gradually increased. For Chinese university students, advancing to higher grades means facing increased exam pressure and multiple stresses such as finding jobs. When confronted with numerous challenges and pressures, university students are prone to developing “learned helplessness” due to repeated setbacks, further weakening the dynamic accumulation of needs (Deci & Ryan, 2012).

Secondly, this study found that FoMO (*β* = 1.360, *p* < 0.001) and depression (*β* = 3.602, *p* < 0.001) are on the rise among college students. Within China’s educational context, senior university students commonly face a phase of multiple overlapping pressures. As academic demands intensify and exam difficulty increases, they often experience persistent anxiety about their academic performance. Simultaneously, the practical task of choosing between employment and further education intensifies their psychological burden. As a major channel for information access, smartphones offer convenience while also creating new psychological burdens. Students feel compelled to maintain constant vigilance to stay updated on job openings and graduate school opportunities, a state that intensifies their FoMO. Under this high-pressure environment, some students repeatedly experience setbacks due to ineffective study methods or poor academic performance, gradually developing feelings of helplessness and inferiority. If such negative emotions accumulate over time, they may trigger depressive disorders (Weinberger et al., 2018). Additionally, on social media, university students are frequently exposed to their peers’ “highlight moments.” These idealized displays can trigger inappropriate upward comparisons, exacerbating feelings of helplessness and inferiority (Beyens et al., 2016).

### 4.2. Direct Effects of Basic Psychological Need Satisfaction on Depression Trends

The findings of the current study indicated that the initial level of basic psychological need satisfaction significantly negatively predicted both the initial level (*β* = −0.236, *p* = 0.031) and developmental rate of depression (*β* = −0.144, *p* < 0.001). Additionally, the developmental rate of basic psychological need satisfaction significantly negatively predicted the developmental rate of depression (*β* = −0.181, *p* = 0.005). These results align with prior research documenting a negative correlation between basic psychological need satisfaction and depression (Xiao & Zheng, 2022). Furthermore, this alignment is consistent with self-determination theory, which posits that when basic psychological needs are sufficiently satisfied, intrinsic motivation and emotion regulation are significantly strengthened, thereby effectively inhibiting depression progression (Deci & Ryan, 2012).

Specifically, individuals with higher initial basic psychological need satisfaction tend to develop positive self-schemas and diminish threat-based appraisals of stressful events, thereby suppressing initial depression levels (Martela & Ryan, 2016). From the conservation of resources theory perspective, individuals with better satisfaction of initial basic psychological needs tend to accumulate psychological resilience during development and can deploy cognitive strategies flexibly when facing stress, thereby effectively interrupting the stress-to-depression pathway (Hobfoll et al., 2018). Additionally, neuroscience research has demonstrated that the satisfaction of basic psychological needs are strongly associated with improved prefrontal cortex (PFC) function, and when individuals’ basic psychological needs are chronically satisfied, the PFC’s ability to regulate the limbic system will be significantly enhanced, which, in turn, reduces hypersensitivity to negative stimuli, thereby decreasing the emergence of depressive moods (Reeve & Lee, 2019).

### 4.3. Longitudinal Mediation of FoMO

This study examined the relationship among basic psychological need satisfaction, FoMO, and depression. The results showed that two indirect paths were established. The first path is basic psychological need satisfaction I–FoMO I–depression I (*β* = −0.132, *p* = 0.007), which suggests that the initial level of basic psychological need satisfaction can influence the initial level of depression by affecting the initial level of FoMO. This may be because individuals with high initial psychological need satisfaction may develop a stable sense of self-efficacy, which helps reduce excessive focus on negative information in social situations, thereby suppressing the initial level of FoMO (Przybylski et al., 2013). In addition, the initial level of FoMO was a significant positive predictor of the initial level of depression, consistent with previous research findings (Ibrahim et al., 2022). College students with high levels of FoMO tend to overemphasize missed social opportunities and positive events experienced by others. This process makes them more prone to persistent negative emotions, thereby providing a potential psychological basis for the development of depression (Elhai et al., 2020). This finding suggests that we need to continue to pay attention to the mental health of university students, especially in their senior years, and provide them with the necessary support and intervention measures to effectively prevent the further deterioration of FoMO and depressive symptoms (Gupta & Sharma, 2021).

The second path is basic psychological need satisfaction I–FoMO I–depression S (*β* = −0.104, *p* = 0.036), which suggests that the initial level of basic psychological need satisfaction not only influences the initial level of FoMO, but also affects the developmental rate of depression. First, in line with the individual–situational interaction theory, the sufficiency of intrinsic resources enables individuals to cope with challenges more effectively when confronting stressful life events (Magnusson, 2015). For individuals with low satisfaction of basic psychological needs, once they encounter setbacks, their psychological systems are less capable of coping with social stress, and they are thus more likely to fall into anxiety, thereby triggering a rapid increase in FoMO (Lai et al., 2016). Second, as FoMO levels rise consistently, individuals’ attention becomes increasingly focused on negative cues such as missed opportunities—a pattern that is likely to elicit negative emotions and disrupt normal regulation functions (Meynadier et al., 2025). According to social comparison theory, individuals experiencing FoMO tend to engage in social comparisons with others, and this state of mind can trigger depression (Park & Park, 2024). Within China’s collectivist cultural context, individual responsibility and reciprocity toward the family are highly emphasized (Wang & Chen, 2010). The interactive features of social media platforms like Weibo and WeChat encourage users to engage in frequent upward social comparison, directly linking these comparisons to family honor. When individuals feel there is a gap between their perceived status on social media and their family’s expectations, amplified by the constant stream of others’ updates, they are highly susceptible to developing negative perceptions. This could be an important risk factor for triggering depression (Reer et al., 2019). This pathway underscores that enhancing basic psychological need satisfaction may mitigate FoMO at its root, offering a key intervention approach for preventing and alleviating depressive symptoms. 

Contrary to the hypotheses, this study did not find that the slope of FoMO mediated the relationship between basic psychological need satisfaction and depression. This may be attributed to several combined factors. First, during early university years, students’ primary task is adapting to the campus environment, with relatively low overall academic and social pressures. This may not have activated significant fluctuations in FoMO, keeping it relatively stable during this period. Consequently, systematic changes affecting depression were difficult to capture in the longitudinal model. Second, as students advance through their academic years, academic pressure and career uncertainty gradually become primary factors influencing depression levels. Since these pressures may occur at different times from the dynamics of FoMO, the FoMO slope pathway failed to reveal a significant mediating effect. Furthermore, as FoMO is a state closely tied to immediate social contexts (Przybylski et al., 2013), its fluctuations often concentrate around specific high-pressure periods (e.g., final exam weeks). This study’s annual measurement interval may have failed to capture such short-term, concentrated fluctuations, potentially overlooking critical FoMO fluctuation periods. Future research could consider multiple measurement points within a single semester to examine FoMO’s dynamic trajectory more precisely. Concurrently, extending the overall tracking period would enable more accurate testing of temporal transmission mechanisms among variables.

This study also found that the relationship among basic psychological need satisfaction, FoMO, and depression is consistent among male and female university students, meaning that there is no gender difference in the longitudinal mediating effect of FoMO on basic psychological need satisfaction and depression. This may be because basic psychological need satisfaction is common to all humans, and when these needs are frustrated, both men and women experience similar negative reactions (Deci & Ryan, 2000). Furthermore, the development of depression is associated with genetic factors, social support, stressful events, or other factors (Herrman et al., 2022), which may weaken the role of gender in this pathway.

### 4.4. Research Implications and Limitations

In terms of theoretical significance, this study introduces FoMO as a mediating variable within the self-determination theory (SDT) framework. Through longitudinal data, it reveals the dynamic psychological mechanism by which the satisfaction of basic psychological needs influences depression. Specifically, the findings validate a core assumption of SDT: when university students’ basic psychological needs remain unmet, it may create a pervasive state of psychological deprivation (Deci & Ryan, 2000), leading to impaired self-regulation and subsequently triggering FoMO and depression. Second, compared to cross-sectional studies, this three-wave longitudinal design more accurately uncovers the temporal relationships among basic psychological need satisfaction, FoMO, and depression. This facilitates a deeper understanding of the developmental trajectories and intrinsic connections among these psychological variables over time. This not only enriches research on the psychological mechanisms underlying depression and development but also provides a theoretical foundation for future studies exploring other mechanisms within the SDT framework.

At the practical level, based on findings from the SDT framework, this study provides valuable practical guidance for university mental health education. Specifically, universities can develop targeted mental health lectures to enhance students’ awareness and capacity to maintain psychological well-being. Curriculum designs should incorporate dedicated modules focusing on autonomy development and competence cultivation. Counselors and psychological practitioners should receive specialized training to improve identification and intervention skills for FoMO. Evidence-based approaches like Cognitive Behavioral Therapy (CBT) should be implemented to help students modify irrational cognitions regarding missed messages. For lower-year undergraduates, targeted academic transition courses can be offered to prevent early frustration. Timely feedback should be provided upon the completion of each task to build and reinforce a sense of competence. For upper-year students, specialized workshops addressing exams and job hunting can dispel the misconception that “one failure means permanent failure,” thereby solidifying their sense of competence. Simultaneously, conducting semester-based surveys on students’ psychological needs and depression could help to proactively identify risks. Schools can help students cultivate self-awareness through science education. When students experience intense FoMO, they should be encouraged to proactively seek practical action plans to fulfill underlying psychological needs. Simultaneously, students must learn to focus on self-positioning, prioritizing personal growth over comparisons with others, thereby alleviating depressive emotions stemming from self-deprecation. This research also helps students to recognize fluctuations in their psychological states, encouraging self-exploration while developing regulatory skills.

This study also has certain limitations. First, while a longitudinal design was employed to partially address the constraints of cross-sectional research, the two-year follow-up period presents limitations: it remains unclear whether this interval is optimal for observing the developmental patterns of these variables, nor does it capture the long-term trajectories of basic psychological need satisfaction and depression among college students over an extended follow-up period. Future research could increase follow-up waves and lengthen the follow-up period to more comprehensively elucidate the relationships between variables. The participants in this study were university students, and the data were collected by the researchers. This may have led some students to complete the questionnaire because they were driven by security needs (such as the desire for excellent grades) or for the purpose of conforming to authority, potentially influencing their perceptions and reports regarding the basic psychological need satisfaction. Future research could employ more rigorous designs and anonymous data collection methods to reduce questionnaire-related biases. Second, this study was restricted to four universities in Anhui Province, with a sample limited to university students, so the representativeness of the findings warrants further examination. Future studies could expand sampling regions to enhance the generalizability of the results. Finally, this study did not examine the differential effects of the three types of basic psychological need satisfaction on depression development. As Deci and Ryan (2000) noted, these needs are distinct and non-interchangeable, and their assessment should be conducted independently when measuring basic psychological need satisfaction. Thus, future research could investigate each need individually to more precisely assess the satisfaction of individuals’ basic psychological needs. Furthermore, a key limitation of this study is that we examined only the unidirectional predictive path from basic psychological need satisfaction to depression. However, depression itself may also diminish individuals’ positive emotional experiences, social interaction motivation, and goal-pursuing behaviors over time (Gronemann et al., 2021), thereby further reducing levels of basic psychological need satisfaction. Future research should employ methods capable of testing bidirectional dynamic relationships (e.g., random intercept cross-lagged panel models) and utilize more frequent time point measurements to uncover the complex reciprocal mechanisms between these variables.

## 5. Conclusions

This study used a longitudinal research method to investigate the relationship among basic psychological need satisfaction, FoMO, and depression. The findings of this study are as follows: over the two-year study period, college students exhibited a decreasing trend in basic psychological need satisfaction, alongside increasing trends in FoMO and depression. The initial level and developmental rate of basic psychological need satisfaction directly predicted the initial level and developmental rate of depression, respectively. The initial level of FoMO mediated the effect of basic psychological need satisfaction on depression. The mediating effect of FoMO in basic psychological need satisfaction on depression did not differ significantly across gender groups of college students. This study contributes to a deeper understanding of the developmental trajectories and intrinsic connections between these psychological variables, offering valuable practical implications for mental health education in universities.

## Figures and Tables

**Table 1:** Descriptive statistics and correlations.

	T1BPNS	T2BPNS	T3BPNS	T1FoMO	T2FoMO	T3FoMO	T1Depression	T2Depression	T3Depression
T1BPNS	1								
T2BPNS	0.243 ***	1							
T3BPNS	0.221 ***	0.394 ***	1						
T1FoMO	−0.129 ***	−0.089 ***	−0.090 *	1					
T2FoMO	−0.171 ***	−0.172 ***	−0.138 ***	0.308 ***	1				
T3FoMO	−0.200 ***	−0.227 ***	−0.265 ***	0.296 ***	0.478 ***	1			
T1Depression	−0.154 ***	−0.096 **	−0.167 ***	0.178 ***	0.096 **	0.129 ***	1		
T2Depression	−0.250 ***	−0.270 ***	−0.249 ***	0.256 ***	0.326 ***	0.378 ***	0.338 ***	1	
T3Depression	−0.297 ***	−0.349 ***	−0.355 ***	0.307 ***	0.359 ***	0.449 ***	0.299 ***	0.669 ***	1
gender	0.016	−0.020	−0.037	0.033	0.011	0.034	0.027	0.019	0.028
M	4.750	4.427	4.093	3.014	3.191	3.354	1.803	1.991	2.166
SD	0.425	0.445	0.514	0.277	0.295	0.369	0.160	0.141	0.177

*Note:* BPNS = basic psychological need satisfaction; FoMO = fear of missing out. T1 = time 1; T2 = time 2; T3 = time 3. Gender is a dummy variable (male = 0, female = 1). *** *p* < 0.001, ** *p* < 0.01, and * *p* < 0.05. The same as below.

**Table 2:** Model fit indices for longitudinal measurement invariance analysis.

Model	χ^2^	*df*	CFI	RMSEA(90% CI)	SRMR	Model Comparison	∆CFI	ΔRMSEA
Basic Psychological Need Satisfaction								
M1:Configural Invariance	575.925	456	0.960	0.019 (0.014, 0.023)	0.050			
M2: Metric Invariance	619.333	492	0.958	0.019 (0.014, 0.023)	0.054	M2-M1	−0.002	0.000
M3: Scalar Invariance	661.156	528	0.956	0.018 (0.013, 0.023)	0.055	M3-M2	−0.002	−0.001
FoMO								
M1: Configural Invariance	70.278	60	0.996	0.015 (0.000, 0.028)	0.019			
M2: Metric Invariance	80.818	74	0.998	0.011 (0.000, 0.024)	0.034	M2-M1	0.002	−0.004
M3: Scalar Invariance	93.215	88	0.998	0.009 (0.000, 0.022)	0.036	M3-M2	0.000	−0.002
Depression								
M1: Configural Invariance	582.461	510	0.976	0.014 (0.007, 0.019)	0.029			
M2: Metric Invariance	603.114	548	0.982	0.012 (0.000, 0.017)	0.032	M2-M1	0.006	−0.002
M3: Scalar Invariance	644.874	586	0.981	0.012 (0.002, 0.017)	0.035	M3-M2	−0.001	0.000

**Table 3:** Model fit of unconditional latent growth model.

Model	χ^2^	*df*	RMSEA	CFI	TLI	Mean (Variance)		r
						Intercept	Slope	
BPNS	0.093	1	0.000	1.000	1.015	90.281 ***	−6.239 ***	0.829
FoMO	0.448	1	0.000	1.000	1.006	24.132 ***	1.36 ***	0.324
Depression	2.252	1	0.041	0.998	0.993	36.142 ***	3.602 ***	0.348

*Note:* *** *p* < 0.001. RMSEA, Root Mean Square Error of Approximation; CFI, Comparative Fit index; TLI, Tucker–Lewis index; r, mean correlation between initial status and rate of change.

**Table 4:** Direct effects in longitudinal mediation analysis.

Direct Paths	*β* [95% CI]	SE	*p*
BPNS intercept → FOMO intercept	−0.370 [−0.517, −0.220]	0.090	<0.001
BPNS intercept → FOMO slope	−0.365 [−0.517, −0.168]	0.106	0.001
BPNS slope → FOMO slope	−0.337 [−0.500, −0.143]	0.110	0.002
BPNS intercept → Depression intercept	−0.306 [−0.466, −0.143]	0.098	0.002
BPNS intercept → Depression slope	−0.348 [−0.519, −0.163]	0.112	0.002
BPNS slope → Depression slope	−0.320 [−0.514, −0.135]	0.118	0.007
FOMO intercept → Depression intercept	0.358 [0.183, 0.513]	0.100	<0.001
FOMO intercept → Depression slope	0.280 [0.109, 0.450]	0.104	0.007
FOMO slope → Depression slope	0.241 [0.034, 0.413]	0.116	0.039

**Table 5:** Indirect effects in longitudinal mediation analysis.

Indirect Paths	*β* [95% CI]	*p*
BPNS intercept → FOMO intercept → Depression intercept	−0.132 [−0.231, −0.070]	0.007
BPNS intercept → FOMO intercept → Depression slope	−0.104 [−0.206, −0.042]	0.036

## Data Availability

The data presented in this study are available on request from the corresponding author. The data are not publicly available due to privacy.

## Abbreviations

The following abbreviations are used in this manuscript:

BPNS	Basic psychological need satisfaction
FoMO	Fear of missing out
